# Health Emergency Research Preparedness: An Analysis of National Pre‑COVID Research Activity and COVID Research Output

**DOI:** 10.5334/aogh.4764

**Published:** 2025-06-13

**Authors:** Peter H. Kilmarx, Shirley Kyere

**Affiliations:** 1 Fogarty International Center, U.S. National Institutes of Health, Bethesda, Maryland, USA

**Keywords:** research capacity, health emergency preparedness, global health research, scientific output metrics

## Abstract

*Background:* Research capacity is a critical element of health emergency preparedness, but metrics are not readily available for many countries. The COVID‑19 pandemic provided an opportunity to use publicly available data to assess correlations between national pre‑pandemic research activity, pandemic research response, and other national socioeconomic characteristics.

*Methods:* National pre‑pandemic (2018–19) research activity was defined as the average of percentile rankings of (1) the average annual number of health science publications in Scopus and (2) the average annual number of clinical trials in the International Clinical Trials Research Platform (ICTRP). National pandemic research response (2020–21) was defined as the average of percentile rankings of (1) average annual number of COVID‑19‑related publications in Scopus and (2) average annual number of COVID‑19‑related clinical trials in ICTRP.

*Findings:* During 2018–19, the median (interquartile range [IQR]) of national annual average health science publications was 415 (108–3,398) and of clinical trials was 21 (4–273). During 2020–21, the median (IQR) of national annual average COVID‑19‑related publications was 85 (18–798) and that of COVID‑19‑related clinical trials was 1.5 (0–11). National COVID‑19‑related research output was strongly correlated with pre‑pandemic research activity (R‑squared 0.89) and much less correlated with Human Development Index (0.26), COVID‑19 case number (0.16), case rate (0.14), gross domestic product (0.11), or population (0.10). In a multivariable linear regression analysis, national pre‑COVID‑19 research activity was the only factor with substantial or statistically significant contribution to explaining variations in COVID‑19‑related research output.

*Interpretation:* National pandemic research responses were most strongly correlated with pre‑pandemic research activity, much more so than with other country characteristics. These findings strongly support global efforts to strengthen research capacity as a critical element of preparedness for health emergencies.

## Introduction

Research capacity is a critical element of health emergency preparedness. Following the large Ebola outbreak in West Africa in the mid‑2010s, the World Bank issued the *Money and Microbes* report, which lamented the “tragic delays” in medical countermeasure development owing to the lack of research capacity in the most‑affected countries and called for sustained efforts to strengthen clinical research capacity in low‑ and middle‑income countries (LMICs) [1]. The Coalition for Epidemic Preparedness Innovations (CEPI), which co‑sponsored the report, subsequently articulated a “100‑day mission” with a global goal of being able to develop “safe, effective, globally accessible vaccines” to new infectious disease threats in as little as 100 days [2].

Since then, the Global Preparedness Monitoring Board, convened by the World Health Organization (WHO) and World Bank, has developed indicators for “capacity building to develop research and development capacities at the regional and national levels” as well as indicators for national assessments for “adequate research capacity deployable at the start of a health emergency” [3]. For example, one indicator asks: “Globally, what is the state of national R&D innovation, development and access to medical countermeasures?” Components of this assessment include: “Do countries globally have adequate research capacity deployable at the start of a health emergency, including genetic sequencing capacity?” and “Do countries globally have independent national ethics review committees with rapid review procedures for research on health emergencies?” However, these have not been fully implemented or comprehensively reported. In addition, the Global Health Security Agenda established a Research & Development Task Force to create metrics to assess national health research capacity [4]. Potential metrics, which could be included in Joint External Evaluations, have been pilot tested in select countries. In 2022, WHO member states adopted resolution WHA75.8 on strengthening clinical trials, and in 2023, they convened the WHO Global Clinical Trials Forum with key stakeholders to advance that goal [5].

In the context of these nascent efforts, the COVID‑19 pandemic provided a unique opportunity to make a global assessment of national‑level research preparedness, productivity, and response. All countries were heavily affected and at least had sufficient numbers and rates of COVID‑19 cases to conduct basic, clinical, and public health research.

We assessed pre‑pandemic research output as a potential predictor of COVID‑19 research productivity. We examined: (1) the feasibility of using easily available data on national research activity as potential indicators of national research capacity, (2) the feasibility of using of easily available data on national disease‑specific research outputs as potential indicators of national pandemic research response, and (3) the extent to which national pre‑pandemic research capacity can predict national pandemic research response in comparison with other national economic and demographic characteristics.

## Methods

We created a metric of each country’s national pre‑pandemic research activity with two elements. One was the percentile ranking of the average of each country’s annual number of health science publications in the Scopus citation database [6] for 2018 and 2019. Publications in Scopus are counted by the country of the authors’ affiliations, and one publication may be counted in more than one country. The second element was the percentile ranking of the average of each country’s annual number of clinical trials in the WHO International Clinical Trials Reporting Platform (ICTRP) [7] for 2018 and 2019. Clinical trials in the ICTRP are counted by the location of participant enrollment sites, and, similarly, one clinical trial may be counted in more than one country. We averaged the two percentile rankings to create an aggregate measure for pre‑COVID‑19 research activity. We limited all analyses to countries with population greater than 100,000 (N = 180) at the time of the analysis.

We created a similar metric of national COVID‑19 research output. We calculated the percentile ranking of the average of each country’s annual number of COVID‑19‑related publications in the Scopus citation database for 2020 and 2021. The search terms were created using synonyms from the COVID‑19 MeSH terms of the National Library of Medicine [8] (Appendix 1). Then we calculated the percentile ranking of the average of each country’s annual number of COVID‑19‑related clinical trials in the ICTRP for 2020 and 2021. A listing of COVID‑19‑related clinical trials was downloaded from the ICTRP website. Then we averaged the two percentile rankings to create an aggregate measure for COVID‑19 research output.

We conducted descriptive univariate analyses of these data and analyzed Pearson’s correlation coefficients to assess the relatedness of the two elements of each of the two metrics. We calculated the R‑squared coefficients of determination and Kendall’s tau rank correlation coefficients of the two metrics—pre‑COVID‑19 research activity and COVID‑19‑related research output. We also analyzed these correlation statistics of COVID‑19 research output with 2022 national population and gross domestic product (GDP) [9], Human Development Index (HDI) in 2022 [10], and national COVID‑19 cumulative case number and rate per capita through December 31, 2021 [11]. We also analyzed these data by the World Bank Income Group [5] and by WHO region [3]. GDP value was missing for two countries, which were excluded from analyses of that variable.

Lastly, we conducted a multivariable linear regression analysis to evaluate predictors of COVID‑19‑related research output during 2020–2021. The predictors examined were pre‑COVID‑19 research activity (2018–19), HDI, COVID‑19 case number, GDP, and population. We computed regression coefficients and assessed their direction and magnitude. The model’s goodness‑of‑fit was evaluated using R‑squared and adjusted R‑squared values; statistical significance was determined at a threshold of p < 0.05.

## Results

In the two years before the COVID‑19 pandemic (2018–19) in the 180 countries with population greater than 100,000, the median of the average annual number of publications was 415, and the median of the average number of clinical trials was 21. These figures were higher in high‑income countries and varied by WHO region, with the highest in Europe and the lowest in Africa and the Western Pacific (Table 1). In the first two years of the COVID‑19 pandemic (2020–21), the median of the average annual number of COVID‑19‑related publications in these countries was 85 and the median of the average number of COVID‑19‑related clinical trials was 1.5 (Table 1). These figures were also higher in high‑income countries and varied by WHO region with the highest in Europe and lowest in Africa and the Western Pacific.

**Table 1:** Medians and interquartile (25th–75th Percentile) ranges of National Metrics of Research outputs, pre‑COVID‑19 and COVID‑19 related, overall and by World Bank Income Group and World Health Organization Region, of countries with population >100,000 (N = 180).

METRIC*	OVERALL	WORLD BANK INCOME GROUP			WHO REGION					
		LOW	MIDDLE	HIGH	AFRICA	AMERICAS	EASTERN MEDITERRANEAN	EUROPE	SOUTH‑EAST ASIA	WESTERN PACIFIC
**Pre‑COVID‑19 (2018–19)**										
**Average annual number of health science publications**	415 (108–3398.5)	122.5 (49–245)	283 (82.0–1455.5)	7411.5 (801–18338.5)	166 (63.5–410)	192 (82–1188)	1417 (222–2498.5)	2325 (476–14682)	816 (41–3136)	179 (28.5–7302.5)
**Average annual number of clinical trials**	21 (4–273)	8 (3.5–13)	12 (2.5–84.5)	357.5 (29.5–1132)	8 (3–17.5)	9.5 (3–72)	26 (7–91)	352 (27.5–829)	27 (7–81)	7.5 (1.5–344.5)
**COVID‑19 (2020–21)**										
**Average annual number of COVID‑19‑related publications**	85 (18–798)	24 (9.5–68)	48.5 (11–422)	808 (164–1886.5)	30 (9–85)	48.5 (11.5–490)	382 (74.5–869.5)	400 (87–1369)	186 (9.5–932)	24 (4–1434.5)
**Average annual number of COVID‑19‑related clinical trials**	1.5 (0–11)	0.5 (0–1.5)	1 (0–5)	12.5 (2–30)	1 (0–2)	1 (0–11.5)	2.5 (0.5–5.5)	7 (1–26)	4 (0–11.5)	0 (0–13)

*Values are the medians and interquartile ranges of the two‑year average numbers of publications or clinical trials in the 180 countries included in the analysis.

There was a strong positive relationship between the national numbers of pre‑COVID publications and clinical trials, with a Pearson’s correlation coefficient of 0.927 (Figure S1). These correlations were moderately to strongly positive across all World Bank income groups and strongly to very strongly positive across all WHO regions (data not shown). Similarly, there was a strong positive relationship between the national numbers of COVID‑19‑related publications and clinical trials, with a Pearson’s correlation coefficient of 0.864 (Figure S2). Also, similarly, these correlations were moderately to strongly positive across all World Bank income groups and moderately to very strongly positive across all WHO regions (data not shown).

As noted in the Methods section, for the main analyses, the raw numbers of publications and clinical trials were converted into rank percentiles and aggregated into aggregate measures, one each for pre‑COVID‑19 research activity and for COVID‑19‑related research output, for each country. Maps of these aggregate measures show the higher research activity and output generally in North America, Western Europe, and East Asia (Figures 1A and 1B). The full data set is available in a Supplemental Data file.

**Figure 1A:**
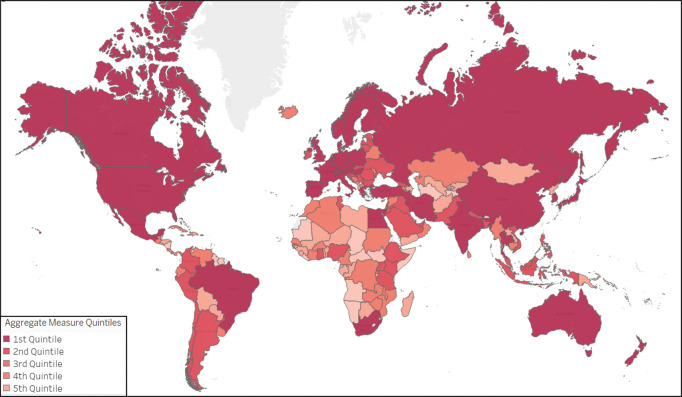
World Map of quintiles of the aggregate measure of pre‑COVID‑19 research activity (number of Health Science publications and clinical trials, 2018–19). A darker hue corresponds to a higher aggregate measure.

**Figure 1B:**
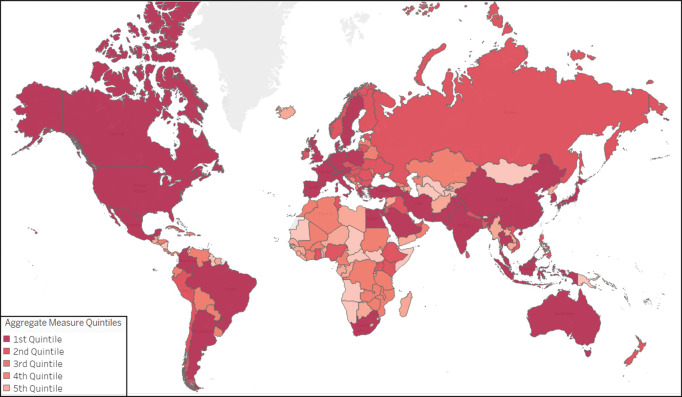
World Map of quintiles of the aggregate measure of COVID‑19‑related research output (number of COVID‑19‑related publications and clinical trials, 2020–21). A darker hue corresponds to a higher aggregate measure.

**Figure 2:**
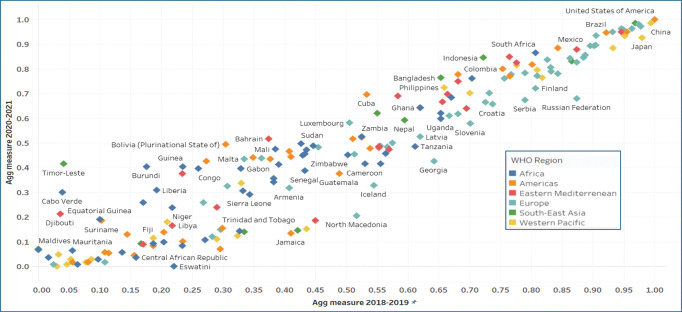
Scatterplot of National Aggregate Metric of pre‑COVID research activity 2018–19 vs. National Aggregate Metric of COVID‑19‑related research output, 2020–21 in countries with population >100,000 (N = 180). R‑squared 0.89; Kendall’s tau 0.81.

The aggregate measure of pre‑COVID‑19 research activity was very strongly positively correlated with the COVID‑19‑related research output, with an R‑squared value of 0.889 and a Kendall’s tau of 0.811 (Figure 2). The other country characteristics we explored (HDI, COVID‑19 case number, COVID‑19 case rate, GDP, and population) were not as strongly correlated with COVID‑19‑related research output (Table 2 and Supplemental Figures S3–S7).

**Table 2:** Correlation statistics of country characteristics with National Aggregate Metric of COVID‑19‑related research output (publications and clinical trials, 2020–21), countries with population >100,000 (N = 180).

COUNTRY CHARACTERISTICS	R‑SQUARED (DESCENDING ORDER)	KENDALL’S TAU
Aggregate metric of pre‑COVID research activity	0.89	0.81
Human Development Index	0.26	0.36
COVID‑19 case number	0.16	0.65
COVID‑19 case rate	0.14	0.32
Gross domestic product (GDP)	0.11	0.68
Population	0.10	0.43

In the multivariable linear regression analysis, taken together, pre‑COVID‑19 research activity (2018‑19), HDI, COVID‑19 case number, GDP, and population accounted for 90.2% of the variance in COVID‑19‑related research output (2020–21). Pre‑COVID‑19 research activity was the only factor with a substantial or statistically significant contribution to explaining variations in COVID‑19‑related research output. Other predictors contributed very little and were not statistically significant (Table 3). Paradoxically, HDI and GDP showed negative trends when accounting for other factors in the model, but these were weak or negligible and not statistically significant.

**Table 3:** Multivariable linear regression analysis of predictors of COVID‑19‑related research output (publications and clinical trials, 2020–21), countries with population >100,000 and complete data (N = 178).

COUNTRY CHARACTERISTICS	UNADJUSTED		ADJUSTED	
	COEFFICIENT	P‑VALUE	COEFFICIENT	P‑VALUE
Aggregate metric of pre‑COVID research activity	1.00	<0.001	1.01	<0.001
Human Development Index	0.98	<0.001	−0.0596	0.33
COVID‑19 case number	2.33 × 10^−8^	<0.001	1.58 × 10^−9^	0.46
Gross domestic product (GDP)	4.49 × 10^−14^	<0.001	−1.81 × 10^−15^	0.72
Population	6.19 × 10^−10^	<0.001	3.76 × 10^−11^	0.52

## Discussion

We used easily available data on national numbers of health science publications and clinical trials as metrics of national research activity, and national numbers of COVID‑19‑related publications and clinical trials as metrics of national pandemic research response. There was a wide range of values, with higher numbers in higher income countries and certain WHO world regions. The national aggregate measure of pre‑pandemic health research activity was very strongly correlated with the national aggregate measure of pandemic research response, much more so than countries’ HDI, burden of COVID‑19 (case number or rate), GDP, or population. These findings strongly support the global efforts to strengthen research capacity as a critical element of preparedness for health emergencies. Notably, in the pandemic, scientists in diverse areas of health research pivoted to conduct critical COVID‑19 studies as part of the emergency response, suggesting that the critical, common elements of clinical research across a range of disease areas can be brought to bear in a health emergency [12].

COVID‑19‑related research outputs were correlated with economic and demographic characteristics such as HDI, GDP, and population, but these were much weaker correlations compared with pre‑pandemic research activity. Future research could examine outliers in the scatterplots as in‑depth case studies of countries that “punch above their weight” with more COVID‑19 research output than expected based on their economic and demographic characteristics.

COVID‑19‑related research outputs were not strongly correlated with either COVID‑19 case numbers (which are confounded with population size) or COVID‑19 case rates. Overall, more COVID‑19 did not necessarily drive more COVID‑19 research; nor, inversely, did more COVID‑19 research seem to correlate with a lower burden of disease.

This previous point raises an important limitation to this analysis. Many clinical trials and publications did not contribute useful evidence that was able to be linked to effective public health or clinical interventions. During the pandemic, it was noted that only about 5% of clinical trials registered in the ICTRP could have been described as randomized and adequately powered [13]. The number of publications and the number of clinical trials are at best crude indicators of research activity and research response to health emergencies. Much more detailed country‑level information is needed, such as the metrics being used by the Global Preparedness Monitoring Board [3] and the Global Health Security Agenda [4], including information on research clinics, laboratory testing, biorepositories, data management, biostatistics, research ethics review, and research regulatory capacity. This information is not readily available for most countries, but it can be inferred from the number of publications and clinical trials for global analyses such as this.

Data on these research outputs may be useful for funders of research capacity strengthening to target resources for countries with lower levels of research capacity [14]. For example, special funding could be made available for north–south or south–south collaborations to build capacity in countries in the lowest quartile or lowest half of these metrics of research activity. These indicators may also be helpful to assess global, regional, and national‑level progress in research capacity strengthening as the number of publications and clinical trials in countries with lower activity increases over time.

We found that pre‑COVID numbers of publications and clinical trials were strongly correlated, as were numbers of COVID‑19‑related publications and clinical trials. This is as expected and validates our combining each of these two elements to create aggregate indicators of pre‑COVID research activity and COVID‑19‑related research output, respectively. The raw data of the number of publications and clinical trials were highly skewed, with very high values for a small number of countries. We therefore used medians rather than means to compare groups in Table 1 and log scales to visualize the data in scatterplots in Figures S1 and S2. We then used percentile rankings in the aggregate measures and the Kendall’s tau statistic as a correlation measure that indicates the strength of the relationship between the two rankings.

The COVID‑19 pandemic provided the opportunity to conduct this analysis, with sufficient case rates and case numbers in almost every country to be able to conduct research and participate in clinical trials. However, in many disease outbreaks, there may be few or no cases in countries with highly developed research capacity, as in the West Africa Ebola outbreak, when it took several months to initiate vaccine clinical trials even after the pathogen and vaccine candidates were identified. As we are unable to predict where the next outbreak may occur, it is important to strengthen clinical research capacity in all countries to ensure national, regional, and global preparedness.

In conclusion, we found that it was a relatively straightforward, reproducible, and feasible process to examine national‑level health science publications and clinical trials, pre‑COVID‑19 and COVID‑19 related, as potential metrics of research capacity and health emergency research response. National COVID‑19‑related research response was very strongly correlated with pre‑COVID‑19 research activity, much more than with other national economic or demographic characteristics. These findings indicate that research capacity strengthening is an important element of preparedness for health emergencies. Efforts to increase capacity, especially in countries with lower levels of research activity, should continue as a global public health priority for the health security of all nations.

## Data Availability

The complete dataset has been made available as a supplemental file.

## Additional Files

The additional files for this article can be found as follows:

Supplementary Figure 1. Scatterplot of National Average Number of Clinical Trials 2018‑19 vs. National Average Number of Publications 2018‑19 in Countries with Population >100,000 (N = 180) (log scales). Pearson’s correlation coefficient 0.86.

Supplementary Figure 1. Scatterplot of National Average Number of COVID‑19‑Related Clinical Trials 2020‑21 vs. National Average Number of COVID‑19‑Related Publications 2020‑21 in Countries with Population >100,000 (N = 180) (log scales). Pearson’s correlation coefficient 0.75.

Supplementary Figure 3. Scatterplot of National Human Development Index vs. National Aggregate Metric of COVID‑19 Related Research Output 2020‑21 in Countries with Population >100,000 (N = 180). R‑squared 0.26; Kendall’s Tau 0.36.

Supplementary Figure 4. Scatterplot of National COVID‑19 Case Number (2020‑21, log scale) vs. National Aggregate Metric of COVID‑19‑Related Research Output 2020‑21 in Countries with Population >100,000 (N = 180). R‑squared 0.16; Kendall’s Tau 0.65.

Supplementary Figure 5. Scatterplot of National COVID‑19 Case Rate per 100,000 Population (2020‑21, log scale) vs. National Aggregate Metric of COVID‑19‑Related Research Output 2020‑21 in Countries with Population >100,000 (N = 180). R‑squared 0.14; Kendall’s Tau 0.32.

Supplementary Figure 6. Scatterplot of National Gross Domestic Product (US$, log scale) vs. National Aggregate Metric of COVID‑19‑Related Research Output 2020‑21 in Countries with Population >100,000 (N = 180). R‑squared 0.11; Kendall’s Tau 0.68.

Supplementary Figure 7. Scatterplot of National Population (log scale) vs. National Aggregate Metric of COVID‑19‑Related Research Output 2020‑21 in Countries with Population >100,000 (N = 180). R‑squared 0.10; Kendall’s Tau 0.43.

Supplementary material. COVID Capacity Analysis data. 23 May 2025.

## Appendix 1: Search Language for a Number of COVID‑19‑Related Publications in Scopus

QUERY: TITLE‑ABS‑KEY (“covid 19” OR “covid‑19” OR “covid19” OR “sars‑cov‑2” OR “sars cov 2” OR “sarscov2” OR “COVID 19” OR “2019‑nCoV Infection” OR “2019 nCoV Infection” OR “2019‑nCoV Infections” OR “Infection, 2019‑nCoV” OR “SARS‑CoV‑2 Infection” OR “Infection, SARS‑CoV‑2” OR “SARS CoV 2 Infection” OR “SARS‑CoV‑2 Infections” OR “2019 Novel Coronavirus Disease” OR “2019 Novel Coronavirus Infection” OR “2019‑nCoV Disease” OR “2019 nCoV Disease” OR “2019‑nCoV Diseases” OR “Disease, 2019‑nCoV” OR “COVID19” OR “Coronavirus Disease 2019” OR “Disease 2019, Coronavirus” OR “Coronavirus Disease‑19” OR “Coronavirus Disease 19” OR “Severe Acute Respiratory Syndrome Coronavirus 2 Infection” OR “COVID‑19 Virus Disease” OR “COVID 19 Virus Disease” OR “COVID‑19 Virus Diseases” OR “Disease, COVID‑19 Virus” OR “Virus Disease, COVID‑19” OR “SARS Coronavirus 2 Infection” OR “COVID‑19 Virus Infection” OR “COVID 19 Virus Infection” OR “COVID‑19 Virus Infections” OR “Infection, COVID‑19 Virus” OR “Virus Infection, COVID‑19” OR “COVID‑19 Pandemic” OR “COVID 19 Pandemic” OR “Pandemic, COVID‑19” OR “COVID‑19 Pandemics” OR “Coronavirus Disease 2019 Virus” OR “Severe Acute Respiratory Syndrome Coronavirus 2” OR “SARS Coronavirus 2” OR “Coronavirus 2, SARS” OR “COVID19 Virus” OR “COVID19 Viruses” OR “Virus, COVID19” OR “Viruses, COVID19” OR “2019‑nCoV” OR “SARS‑CoV‑2 Virus” OR “SARS CoV 2 Virus” OR “SARS‑CoV‑2 Viruses” OR “Virus, SARS‑CoV‑2” OR “COVID‑19 Virus” OR “COVID 19 Virus” OR “COVID‑19 Viruses” OR “Virus, COVID‑19” OR “2019 Novel Coronavirus” OR “2019 Novel Coronaviruses” OR “Coronavirus, 2019 Novel” OR “Novel Coronavirus, 2019”).
