# Supplementary material for: Health Emergency Research Preparedness: An Analysis of National Pre‑COVID Research Activity and COVID Research Output
Source: Ann Glob Health. 2025 Jun 13;91(1):33. doi: 10.5334/aogh.4764 (PMC12171802; doi:10.5334/aogh.4764)
Supplement: Supplementary Figure 1. — Scatterplot of National Average Number of COVID‑19‑Related Clinical Trials 2020‑21 vs. National Average Number of COVID‑19‑Related Publications 2020‑21 in Countries with Population >100,000 (N = 180) (log scales). Pearson’s correlation coefficient 0.75. [file agh-91-1-4764-s2.pdf]

Fig S2

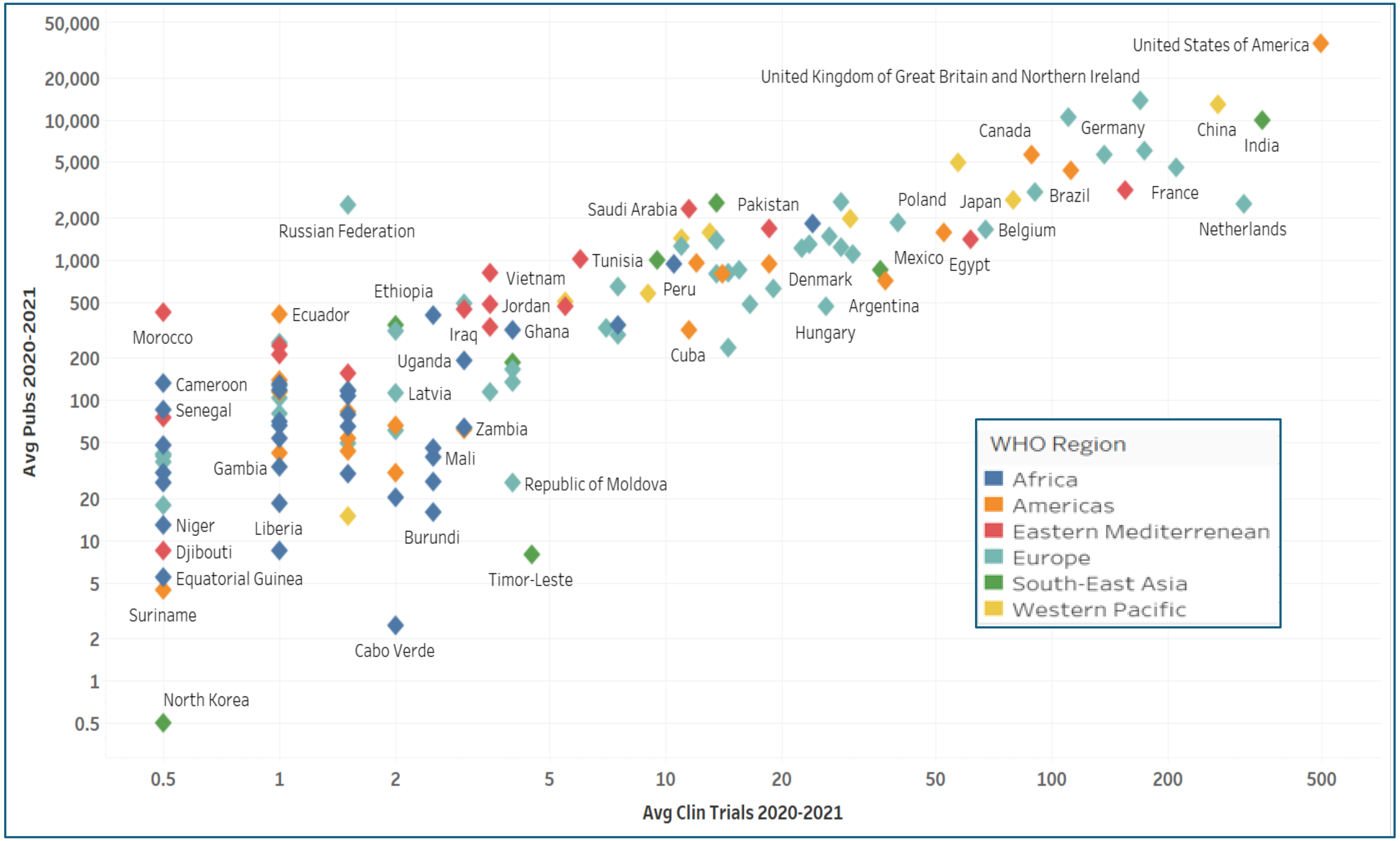

S2. Scatterplot of National Average Number of COVID-19-Related Clinical Trials 2020-21 vs. National Average Number of COVID-19-Related Publications 2020-21 in Countries with Population >100,000 (N = 180) (log scales). Pearson's correlation coefficient 0.75.
