# Supplementary material for: Health Emergency Research Preparedness: An Analysis of National Pre‑COVID Research Activity and COVID Research Output
Source: Ann Glob Health. 2025 Jun 13;91(1):33. doi: 10.5334/aogh.4764 (PMC12171802; doi:10.5334/aogh.4764)
Supplement: Supplementary Figure 3. — Scatterplot of National Human Development Index vs. National Aggregate Metric of COVID‑19 Related Research Output 2020‑21 in Countries with Population >100,000 (N = 180). R‑squared 0.26; Kendall’s Tau 0.36. [file agh-91-1-4764-s3.pdf]

Fig S3

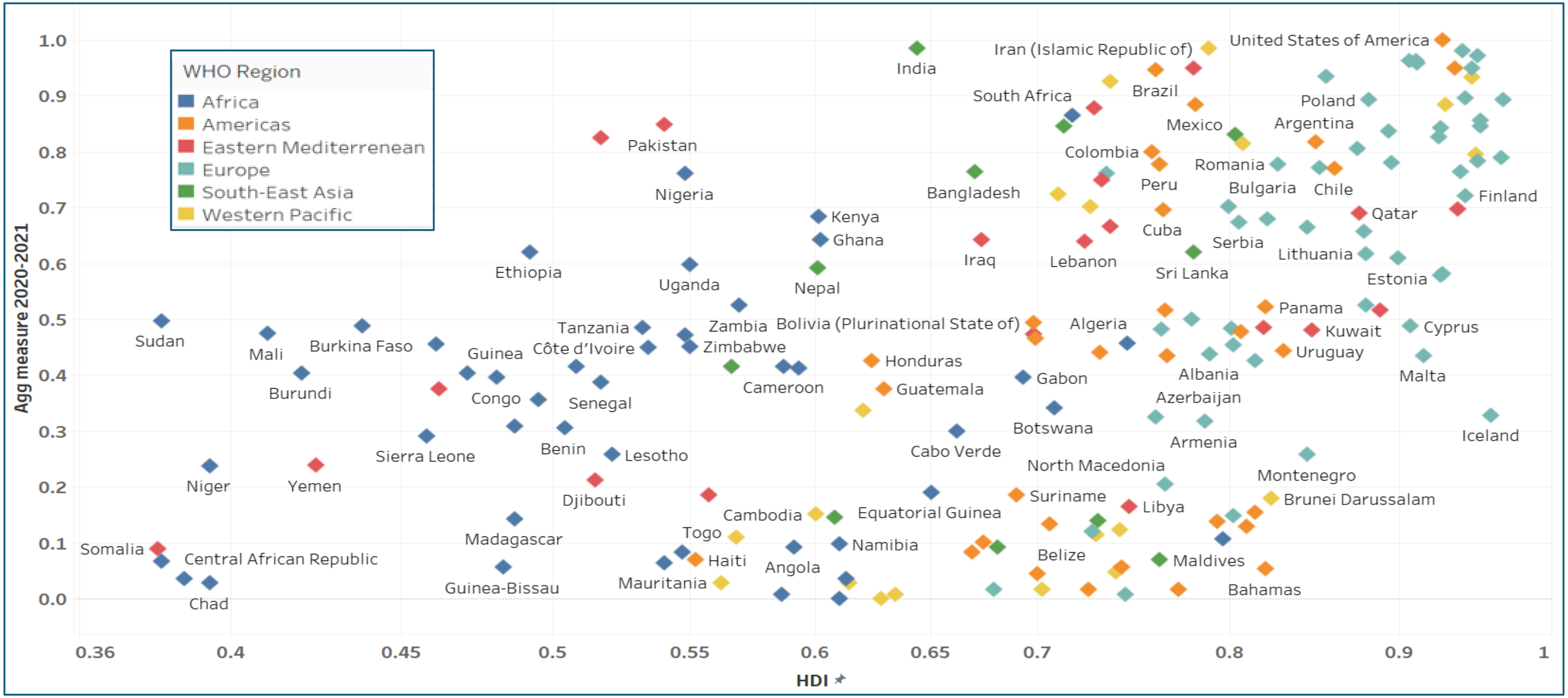

S3. Scatterplot of National Human Development Index vs. National Aggregate Metric of COVID-19 Related Research Output 2020-21 in Countries with Population >100,000 (N = 180). R-squared 0.26; Kendall's Tau 0.36.
