# Supplementary material for: Health Emergency Research Preparedness: An Analysis of National Pre‑COVID Research Activity and COVID Research Output
Source: Ann Glob Health. 2025 Jun 13;91(1):33. doi: 10.5334/aogh.4764 (PMC12171802; doi:10.5334/aogh.4764)
Supplement: Supplementary Figure 4. — Scatterplot of National COVID‑19 Case Number (2020‑21, log scale) vs. National Aggregate Metric of COVID‑19‑Related Research Output 2020‑21 in Countries with Population >100,000 (N = 180). R‑squared 0.16; Kendall’s Tau 0.65. [file agh-91-1-4764-s4.pdf]

Fig S4

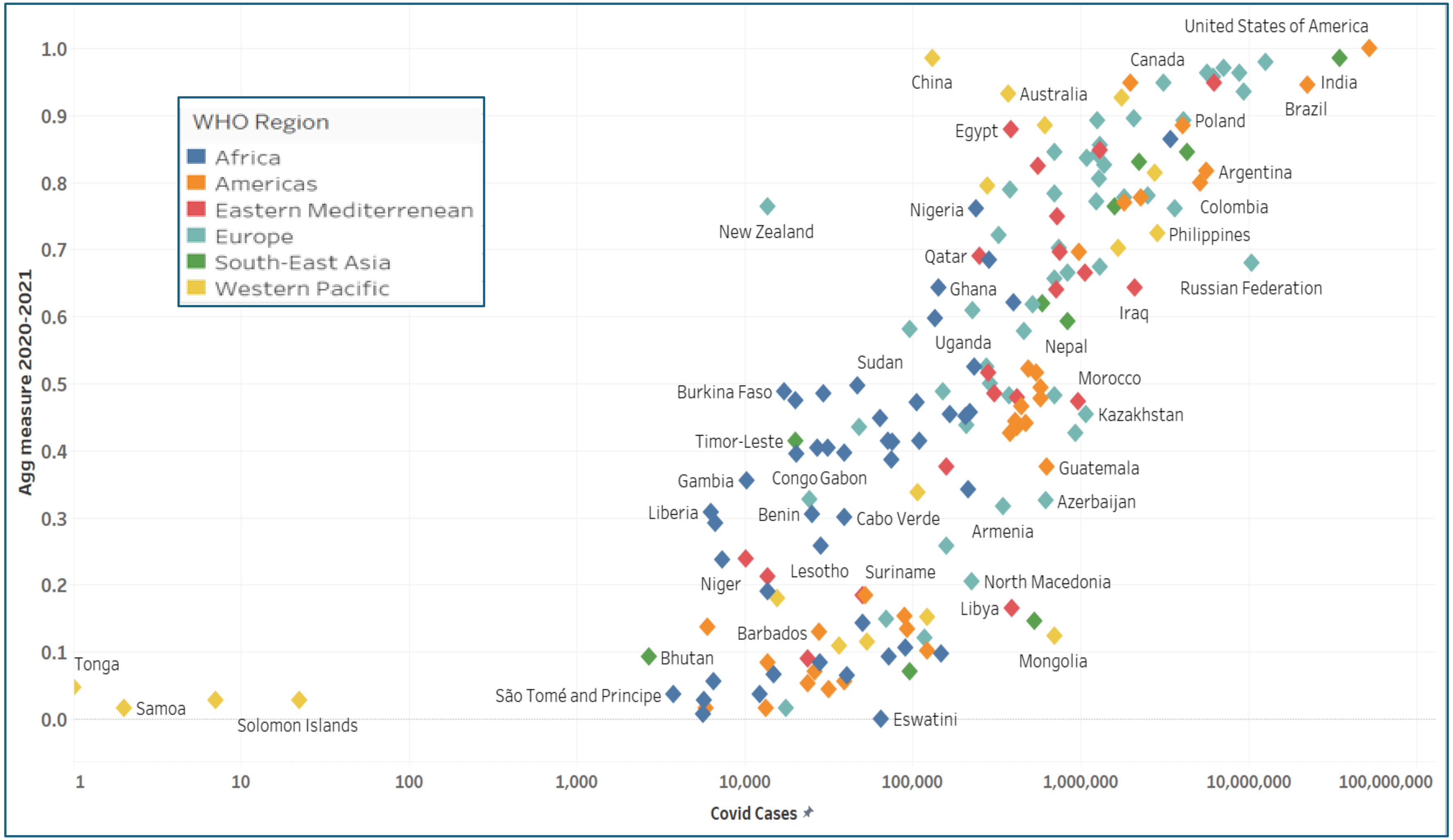

S4. Scatterplot of National COVID-19 Case Number (2020-21, log scale) vs. National Aggregate Metric of COVID-19-Related Research Output 2020-21 in Countries with Population >100,000 (N = 180). R-squared 0.16; Kendall's Tau 0.65.
