# Supplementary figures and images for: Health Emergency Research Preparedness: An Analysis of National Pre‑COVID Research Activity and COVID Research Output
Source: Ann Glob Health. 2025 Jun 13;91(1):33. doi: 10.5334/aogh.4764 (PMC12171802; doi:10.5334/aogh.4764)

Fig S7

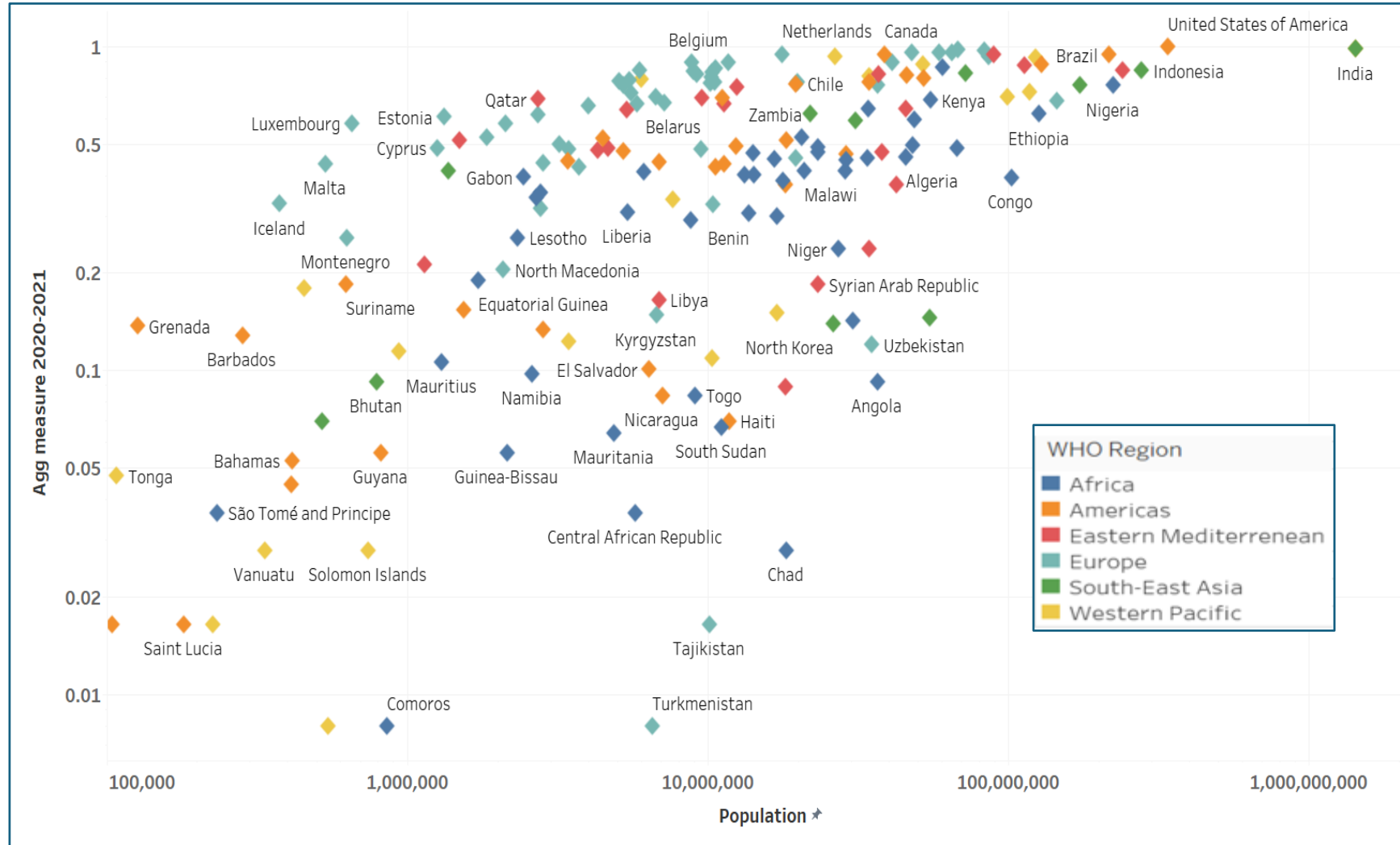

Supplement: Supplementary Figure 7. — Scatterplot of National Population (log scale) vs. National Aggregate Metric of COVID‑19‑Related Research Output 2020‑21 in Countries with Population >100,000 (N = 180). R‑squared 0.10; Kendall’s Tau 0.43. [file agh-91-1-4764-s7.pdf]
